# Treatment Outcomes for Patients Undergoing Hemodialysis with Chronic Hepatitis C on the Sofosbuvir and Daclatasvir Regimen

**DOI:** 10.7759/cureus.5702

**Published:** 2019-09-19

**Authors:** Nazish Butt, Amanullah Abbasi, M Ali Khan, Muhammad Ali, Ghulam B Mahesar, Farhan Haleem, Abdul Manan

**Affiliations:** 1 Gastroenterology, Jinnah Postgraduate Medical Centre, Karachi, PAK; 2 Internal Medicine, Dow University of Health Sciences, Karachi, PAK; 3 Nephrology, Jinnah Postgraduate Medical Centre, Karachi, PAK; 4 Internal Medicine, Jinnah Postgraduate Medical Centre, Karachi, PAK

**Keywords:** hep c, sofosbuvir, daclatasvir, hemodialysis, end-stage-renal-disease

## Abstract

Background

Hepatitis C (HCV) infection is the most commonly acquired infection for patients on hemodialysis and is associated with significant morbidity and disease progression. Direct-acting antivirals (DAAs) have revolutionized the management of HCV. However, limited data exist regarding their efficacy in end-stage renal disease (ESRD), especially for patients on dialysis in South Asia.

Aims

To evaluate the treatment outcomes of patients undergoing hemodialysis with chronic hepatitis C (CHC) on the sofosbuvir (SOF) and daclatasvir (DAC) regimen.

Materials and methods

All patients who were 18 years or older, diagnosed cases of chronic kidney disease (stage V), and undergoing maintenance hemodialysis were inducted into this study. Active HCV infection was demonstrated by polymerase chain reaction (PCR) HCV ribonucleic acid (RNA) (qualitative). All patients were then treated with a double regimen of SOF (400 mg once daily) and DAC (60 mg once daily) taken per oral for 12 weeks. Response to treatment was assessed at four, 12, and 52 weeks.

Results

A total of 31 out of 80 patients were inducted into the study over two years. The prevalence of HCV in hemodialysis patients was 38.75%. Sustained virological response (SVR) was achieved by 27 (87.09%) patients at one year. Four (12.90%) patients had a relapse of HCV. There was no deterioration of hepatological status in any of the patients. Overall survival at one year was 93.54%.

Conclusion

HCV is highly prevalent in patients undergoing hemodialysis. Prompt treatment with SOF and DAC demonstrates a good response, with negligible side effects.

## Introduction

Chronic kidney disease (CKD) in South Asia is highly prevalent, notably in developing countries. The prevalence of CKD in Pakistan is estimated to be around 23% [1]; most of these estimates are from studies conducted within the urban areas, especially urban Karachi. A large number of these patients eventually require hemodialysis for the maintenance of normal renal function.

While numbers vary from 60%-82% of patients with CKD requiring dialysis, increasing age and comorbidities are associated with a higher and earlier need for dialysis [2]. Once CKD patients are put on hemodialysis, they are at an increased risk of complications such as myocardial infarction, iron deficiency anemia, acquired infections, fistula formation, and osteoporosis, to name a few [3-4].

Acquired infections are common and associated with increased morbidity and mortality. Chronic hepatitis C (CHC) is the most commonly transmitted infection in CKD patients on hemodialysis; it is also one of the causes of CKD itself [5]. The natural course of CHC with CKD isn’t simple and both diseases affect each other. CHC is associated with a greater risk of cardiovascular and liver-related mortality in patients on dialysis [6].

Historically, treatment for CHC was arduous even for patients without CKD, even more so for patients on maintenance hemodialysis. With polyethylene glycol (PEG)-interferon, only 39% of patients exhibited viral eradication. Overall, a sustained virological response was not achieved even if it was tolerated [7]. Direct-acting antivirals (DAAs) are a newly developed class of drugs that have shown great efficacy in treating CHC.

For patients on hemodialysis taking DAAs, viral elimination rates of as high as 99% have been seen, with little or no adverse events [8], mostly for genotypes 1, 4, and 6. However, little data is available on the treatment outcomes with sofosbuvir and daclatasvir. Even less data is available on genotype 3 in this aspect. Couple the above facts with respect to Pakistan and almost no data is available at all, something we look to rectify here.

## Materials and methods

Data were collected from Ward 23, Gastroenterology, and Ward 22, Nephrology, Jinnah Postgraduate Medical Centre, Karachi. This study was held from January 2017 to December 2018. Approval from the ethical committee/board of the institute had been taken. All information was kept confidential. Written consent was taken from all patients.

Initial screening for HCV was done via the enzyme-linked immunosorbent assay (ELISA) test for the HCV antibody. Only patients that had a positive result were further tested. Patients were made fully aware of the procedures that were being carried out and the regimen used to treat chronic hepatitis C (CHC) was provided free of cost.

Inclusions criteria

Patients of either gender, who were 18 years or older and met the following criteria, were inducted into the study:

1. A positive qualitative polymerase chain reaction (PCR) result and genotype detection indicative of an active HCV infection.

2. Already diagnosed cases of HCV, with proven active replication.

3. Patients who were undergoing maintenance hemodialysis due to end-stage renal disease (ESRD), defined as a glomerular filtration rate (GFR) of less than 30 mL/min/1.73 m^2^, which required weekly hemodialysis sessions to maintain renal functions.

Exclusion criteria

1. Patients with decompensated liver disease or compensated cirrhotics (on the basis of clinical, history, and lab findings)

2. Patients with terminal or metastatic malignancy

3. HIV co-infected patients

4. HBV co-infected patients

5. Intravenous (I/V) drug abusers

6. Other co-infections, such as Mycobacterium tuberculosis, fungal infections, or other opportunistic infections

7. Alcoholics

Primary outcome

The primary outcome was to achieve viral elimination from the serum. PCR was done at selected intervals to evaluate the response to treatment. Rapid virological response (RVR) was seen at four weeks since starting treatment, end of treatment response (ETR) was seen at 12 weeks, and sustained virological response (SVR) was evaluated at 52 weeks since starting treatment.

Secondary outcome

The eventual course of ESRD and liver status at one year was also assessed. Any deterioration in the liver function and disease progression for both CKD and CHC were recorded.

Drug regimen

Sofosbuvir (SOF) is an inhibitor of the NS5B nonstructural protein and daclatasvir (DAC) is an inhibitor of NS5A nonstructural protein. These phosphoproteins play an important role in hepatitis C replication. For patients without cirrhosis, DAC was used in combination with SOF according to the guidelines of that time. We used the following regimen:

· DAC 60 mg once daily per oral + SOF 400 mg once daily per oral for 12 weeks

Note: The medicine was given OFF-LABEL to all patients free of cost. At the time of this study, other newer DAAs, such as Ombitasvir, Grazoprevir, Paritaprevir with Ritonivir, etc., were neither licensed nor available for use in Pakistan (even for clinical trials).

Laboratorical analysis

All baseline labs were done at period intervals of four, 12, and 52 weeks after induction into the study. These included complete blood count (CBC), prothrombin time (PT), international normalized ratio (INR), urea and creatinine levels, with serum electrolytes. Ultrasound of the abdomen was also carried out. All this was free of cost for the patients.

Data analysis and sampling technique

The nonprobability convenient sampling technique was used. Statistical Package for the Social Sciences (SPSS) version 21.0 software (SPSS Inc., Chicago, IL, USA) was employed.

## Results

Of the 80 patients that were screened over two years, 25 patients had a positive result for HCV antibody. Six patients were previously diagnosed cases of HCV with active infection were also included. Incidentally, all 31 also had positive PCR as well, putting the prevalence at 38.75%.

Demographic and baseline characteristics

Patients were predominantly female, middle-aged, and housewives. Demographic details are shown in Table 1. Patients had low mean hemoglobin (Hb) as one would expect in CKD, no other abnormality could be seen. Baseline characteristics are summarized in Table 2.

**Table 1:** Demographics

	(N=31)
Age (mean)	36.52±10.90 years
Gender	
Male	11 (35.48%)
Female	20 (64.51%)
Marital Status	
Married	26 (83.87%)
Not Married	5 (16.12%)
Occupation	
Housewife	20 (64.51%)
Laborer	04 (12.90%)
Shopkeeper	03 (9.67%)
Other	04 (12.90%)

**Table 2:** Baseline characteristics

	(N=31)
Age (mean)	36.52±10.90 years
Gender	
Male	11 (35.48%)
Female	20 (64.51%)
Marital Status	
Married	26 (83.87%)
Not Married	5 (16.12%)
Occupation	
Housewife	20 (64.51%)
Laborer	04 (12.90%)
Shopkeeper	03 (9.67%)
Other	04 (12.90%)

Renal, electrolyte, and dialysis profile

Creatinine and urea understandably were on the higher side with patients requiring a median of three dialysis sessions per week. Hypertension was the most common cause of CKD requiring dialysis. The renal and electrolyte profile is given in Table 3 and the dialysis profile in Table 4.

**Table 3:** Renal and electrolyte profile

	(N=31) Mean
Creatinine	7.82±2.75
Urea	117.87±36.37
Sodium	136.00±4.11
Potassium	4.37±0.82
Bicarbonate	23.53±2.41
Phosphate	6.96±1.42
Calcium	7.78±0.77

**Table 4:** Dialysis profile

	(N=31)
Cause of Chronic Kidney Disease/Dialysis	
Hypertension	12 (38.7%)
Miscellaneous*	07 (22.6%)
Bilateral Small Size Kidney Disease (BSSKD)	06 (19.4%)
Diabetes	04 (12.9%)
Renal Stone Disease	02 (6.5%)
Duration of dialysis per week (median)	03/week
Duration of ongoing dialysis (mean)	3.37±1.54 years
*These include chronic tubulointerstitial nephritis, multiple myeloma, pregnancy-induced renal disease, amyloidosis, and echogenic kidneys.	

Liver and serological profile

A majority of the patients were genotype 3 (GT-3) and treatment naïve. The liver function test was representative of chronic liver disease without decompensation or cirrhosis. Any alterations seen could only be attributed to CKD rather than CHC. Liver and viral profiles are summarized in Tables 5-6.

**Table 5:** Liver biochemistries

	(N=31)
Alanine Aminotransferase (ALT)	37.25±47.10
Aspartate Aminotransferase (AST)	39.57±39.91
Gamma-Glutamyl Transferase (GGT)	51.20±23.03
Alkaline Phosphatase (ALP)	540.64±712.29
Albumin	3.27±0.53

**Table 6:** Viral/HCV/serological and treatment status HCV: Hepatitis C Virus; PEG-Interferon: Pegylated Interferon

	(N=31)
Time since diagnosis of HCV (median)	9 months
HCV genotype	
Genotype 1	10(32.30%)
Genotype 3	21(67.70%)
Treatment status	
Treatment naïve	25 (80.6%)
Treatment experienced	6 (19.4%)
PEG-interferon	3 (50.0%)
Conventional interferon	3 (50.0%)

Primary outcome with subgroup analysis

A good number (87.09%) of patients achieved a sustained vIrological response (SVR). The response was equally distributed through genotype, etiology, and treatment naïve vs. experienced groups. There were only four relapses in the entire study, and they were found to be statistically insignificant overall. The primary outcome and subgroup analysis are shown in Table 7 and Table 8, respectively.

**Table 7:** Primary outcome

(N=31)	Rapid virological response (04 weeks)	Early virological response (12 weeks)	Sustained virological response (52 weeks)
Achieved	29 (93.5%)	29 (93.5%)	27 (87.09%)
Not achieved	2 (6.5%)	2 (6.5%)	4 (12.9%)

**Table 8:** Subgroup analysis CKD: Chronic Kidney Disease, BSSKD: Bilateral Small Size Kidney Disease

Cause of CKD	SVR	Genotype 1	Genotype 3
Hypertension	Achieved	2	10
	Not achieved	Nil	Nil
Miscellaneous	Achieved	3	3
	Not achieved	Nil	1
BSSKD	Achieved	Nil	4
	Not achieved	2	Nil
Diabetes	Achieved	2	1
	Not achieved	1	Nil
Stones	Achieved	Nil	2
	Not achieved	Nil	0
Treatment naive	Achieved	7	15
	Not achieved	2	1
Treatment experienced	Achieved	Nil	5
	Not achieved	1	Nil
Overall	Achieved	7 (70.0%)	10 (90.90%)
	Not achieved	3 (30.0%)	1 (9.10%)

Secondary outcome

There was no development of overt cirrhosis or decompensation of liver function at all. Twenty-seven patients continued on maintenance hemodialysis after achieving SVR. Two patients had a relapse of HCV although no deterioration in renal function was seen until the completion of the study. Two patients succumbed to ESRD and its natural disease progression. Overall survival was 93.54% at one year. Table 9 summarizes the secondary outcomes.

**Table 9:** Secondary outcomes CKD: Chronic Kidney Disease, BSSKD: Bilateral Small Size Kidney Disease

Cause of CKD	SVR	Genotype 1	Genotype 3
Hypertension	Achieved	2	10
	Not achieved	Nil	Nil
Miscellaneous	Achieved	3	3
	Not achieved	Nil	1
BSSKD	Achieved	Nil	4
	Not achieved	2	Nil
Diabetes	Achieved	2	1
	Not achieved	1	Nil
Stones	Achieved	Nil	2
	Not achieved	Nil	0
Treatment naive	Achieved	7	15
	Not achieved	2	1
Treatment experienced	Achieved	Nil	5
	Not achieved	1	Nil
Overall	Achieved	7 (70.0%)	10 (90.90%)
	Not achieved	3 (30.0%)	1 (9.10%)

## Discussion

There is little data available on HCV infected patients with CKD on maintenance hemodialysis in Pakistan. Most studies that are present tend to look at the prevalence, risk factors, genotypes, and treatment outcomes with PEG-interferon rather than DAAs. No data was available on treatment with DAAs in this group even five years after first approval of SOF. Thus, we were breaking new ground when we started this study.

The prevalence of active HCV infection for patients on maintenance hemodialysis was found to be 38.75%. This is much higher than what was stated by Khokar N et al. [9]; the prevalence of HCV was reported to be 23.7%. However, Gul A et al. [10] reported the prevalence to be 68% in similar patients. Other studies have estimated the prevalence to be as high as 73%.

The limited data that is available in Pakistan points to a high prevalence of HCV in patients going under hemodialysis. Therefore, vigorous screening protocols should be instituted as early detection before the development of cirrhosis and treatment with DAAs confer greatly reduced morbidity and mortality as we discuss below.

Most patients were female and married (housewives). No correlation between CKD and HCV acquisition to the occupation of the patients could be made. The relatively “young” median age of our patients can be attributed to early causes of CKD such as pregnancy-induced renal disease, bilateral small size kidney disease (BSSKD), and echogenic kidneys.

CKD is associated with iron deficiency anemia and low mean corpuscular volume (MCV). But our patients were already taking preventive measures against this, thus Hb and MCV were fairly well-controlled. No immunocompromised patients were inducted into the study and neither were any cirrhotics, as such white blood cells and platelets were within normal ranges.

With a median time since the start of dialysis of three years, almost all patients were undergoing the thrice-weekly dialysis routine. Since we could not identify clear-cut risk factors for the transmission of HCV with the exception of hemodialysis itself, it is the opinion of the authors that most patients were infected after the initiation of maintenance hemodialysis. This also corresponds to the time of diagnosis of HCV, i.e. nine months median.

Hypertension and diabetes constituted more than half of all the causes of CKD in this study. These are associated with a high risk of CKD developing ESRD requiring hemodialysis [11]. BSSKD, yet another disease that affects primarily a younger age group, was also prevalent. Singular cases of amyloidosis and chronic tubulointerstitial nephritis were also seen.

The most common subtype for HCV is genotype 3 in Pakistan and this was reflected in our study, with 67.70% patients exhibiting the said genotype. Only six patients had previously taken medications for HCV, to no avail (see Table 6). Both PEG-interferon and conventional interferon were prescribed for one year in all six patients. These treatments were carried out before the licensed used of DAAs in Pakistan.

Since all patients were CHC and noncirrhotic, the liver function tests (LFTs) are representative of this. The raised alkaline phosphatase (AP) could not be attributed to CHC but to CKD. For the most part, patients with CKD have AP in the nonpathological ranges with higher than normal means [12]. In our study, this equation was disrupted by the two cases of multiple myeloma, which raised the mean significantly.

The overall SVR achieved at one year in our study was 87.09%. Previously, SVRs ranging from 58% to 100% have been reported using the SOF-based regimen in patients undergoing hemodialysis [13]. These studies did not use DAC as the second drug. The treatment outcome for HCV GT-3 was better than GT-1; the SVR achieved for GT-1 was similar to that reported for the region [14-15], whereby worsening CKD is associated with lower SVRs and viral elimination.

Good results have been seen with DAAs for both treatment experience and naïve patients. This was the case in our study, with both groups showing similar SVR rates. The difference was statistically nonsignificant. The highest number of relapses was seen in the BSSKD group. One-third of the patients with BSSKD relapsed at one year; all were GT-1, which is also the most probable cause of this phenomenon.

Survival at one year was 93.54%. Despite having active HCV and CKD for a considerable time, our patients did not show any deterioration in liver function. This is not to say that both diseases do not affect each other in a bidirectional manner. This simply indicates that if treated earlier, HCV elimination reduces mortality considerably. Patients that were found to be cirrhotics or decompensated were not inducted into the study; this too contributed to the high SVR achieved and survival rates.

Of the two patients that passed away, both had relapsed at one year. Still, there was no deterioration of the liver function to the decompensated state. The cause of death in both cases was the natural progression of ESRD. Two other patients relapsed as well and continued their CKD treatment while awaiting approval for newer DAAs.

Throughout the study, no major side effects were noted with the use of DAAs. No patients were lost to follow-up nor did they fall back on their nephrological consults and dialysis sessions. Few shortcomings were associated with this study. The sample size was too small, but this did constitute a major bulk of CKD patients requiring hemodialysis in our center. Patients were only followed up for one year with respect to their virological response. Whether HCV relapse occurred after that or not was not analyzed.

Furthermore, the effect and interactions of drugs already being taken by the patients with the DAAs were not evaluated either. IL28 B genotyping was not done. While it was made sure that patients were taking adequate calories, a complete nutritional assessment was not carried out throughout the study.

## Conclusions

We conclude that the burden of HCV in patients on hemodialysis is high. Treatment before the development of cirrhosis reduces mortality. SOF plus DAC is well-tolerated and effective in the treatment of CHC in these patients; it is also associated with an improved survival rate. Until newer DAAs are made available in Pakistan, we recommend this regimen.
